# Factors influencing HIV risk-taking behaviours amongst textile factory workers living with HIV in Lesotho

**DOI:** 10.11604/pamj.2019.33.166.18961

**Published:** 2019-07-04

**Authors:** Refiloe Stephania Mabathoana, Chantel Van Wyk, Anthonio Oladele Adefuye

**Affiliations:** 1 Division of Health Sciences Education, Office of the Dean, Faculty of Health Sciences, University of the Free State, PO Box 339, Bloemfontein 9300, South Africa

**Keywords:** Lesotho, textile factory workers, HIV, risk-taking behaviours

## Abstract

With its number of employees ranging from 45,310 to 46,000, the textile and apparel industry is the main private sector employer of labour in Lesotho. It has been reported that a third (an estimated 34%) of these workers are living with HIV. There is perception that textile factory workers living with HIV (TFWLWH) in Lesotho indulge in HIV risk-taking behaviours. However, no study has yet investigated or documented factors that influence risk-taking behaviours amongst these workers. Transmitting the disease to others, treatment complications and death consequent to HIV reinfection are complications associated with HIV risk-taking behaviours by seropositive individuals. Using an in-depth, face-to-face, semi-structured interview, this study obtained the perspectives of ten factory workers from three randomly selected textile factories in Maseru, Lesotho on factors that influence HIV-risk taking behaviour amongst TFWLWH in Lesotho. Analysis of the comments given by workers revealed four core themes, namely, peer pressure, communication, cultural norms and societal norms. Determining the predictors of HIV risk-taking behaviours amongst these workers will inform both present and future interventions aimed at supporting textile factory workers living with HIV in Lesotho. This supports the need for continued research to identify HIV risk-taking behaviours by people living with HIV countrywide, to decrease the incidence of new infections and complications arising from reinfection.

## Introduction

Since recording its first case of AIDS in 1986, the number of HIV infections in Lesotho has increased exponentially [1]. With an adult prevalence rate of 25% [2], Lesotho has one of the highest HIV burdens in the world [3]. The worst affected groups include sex workers (79.1% prevalence), textile factory workers (42.7% prevalence), men who have sex with men (32.9% prevalence), prison inmates (31% prevalence), and pregnant women (25.9% prevalence) [4]. With an estimated 45,310 to 46,000 employees [5, 6], of whom the majority are young women and a third (an estimated 34%) are HIV positive [7], the textile and apparel industry is the main private sector employer of labour in Lesotho [5]. In order to lessen the HIV/AIDS burden amongst workers in the textile industry, the ComMark Trust-funded by the UK Department for International Development (DFID) in 2005-invented the Apparel Lesotho Alliance to Fight AIDS (ALAFA) project, to provide support for HIV-positive workers in the textile and apparel industry [8]. There is perception that TFWLWH in Lesotho indulge in HIV risk-taking behaviours. However, no study has yet investigated factors that influence indulging in HIV risk-taking behaviours by these workers. Using an in-depth, face-to-face, semi-structured interview, this study obtained the perspectives of ten factory workers on factors that influence the indulgence in HIV-risk taking behaviour by TFWLWH in Lesotho. Indulging in HIV risk-taking can lead to HIV reinfection [9], increased viral load and disease progression [10], and increased chance of acquiring resistant strains [11]. Determining the predictors of HIV risk-taking behaviours by these workers will inform both present and future interventions aimed at supporting TFWLWH in Lesotho.

## Methods

This research was designed as a qualitative study that made use of an in-depth, face-to-face, semi-structured interview to obtain qualitative data.

**In-depth, face-to-face, semi-structured interviews:** participants were interviewed using a pretested schedule.

**Study settings:** study was conducted at the premises of three randomly selected textile factories (names withheld) located in the Maseru district of Lesotho.

**Study participants:** participants in this study were selected using the following eligibility criteria: textile factory workers belonging to the ALAFA support group; male or female factory workers, 18 years or older; workers infected with HIV and receiving antiretroviral treatment.

**Unit of analysis:** a purposive and voluntary sampling technique was used for this study. Eligible individuals who were willing to participate were invited and briefed during an introductory meeting where confidentiality and the voluntary nature of participation was ensured. Nine volunteers (three in each factory) participated in the study.

**Exploratory interview:** an exploratory interview was conducted to test the suitability of the questions in the interview guide. This interview was conducted with one eligible participant, and no changes to the interview schedule were indicated after administration. Findings from the exploratory interview was included in the dataset.

**Data collection:** interviews were conducted according to a schedule of predetermined appointments in a private, quiet room, in the presence of an independent observer and each session lasted about 45-60 minutes. All interviews were conducted in the local language of the respondents (Sesotho) and were audio recorded. Field notes were taken to enable reflection on details of each interview. Immediately after each interview session, all notes and audio recordings were reviewed. This resulted in a process of concurrent data collection, preliminary analysis, reflection and progressive focusing.

**Data analysis and interpretation:** the recordings were transcribed and translated to English by one of the researchers and quality assured by the independent observer. Thematic analysis formed the cornerstone of the analysis, and was done using NVivo 12 software (QSR International Pty Ltd, Australia). Specific attention was given to patterns and emerging themes.

**Ethical considerations:** the Health Sciences Research Ethics Committee of the University of the Free State (ECUFS Nr 158/2014) approved this study.

## Results

**Demographic information of participants:** ten interviews were conducted (7 women and 3 men). Participants were between the ages of 18 and 31 years.

**Factors that influence engaging in HIV risk-taking behaviours by workers:** analysis of the comments given by the workers revealed four core themes, namely, peer pressure, communication, cultural norms, and societal norms. Each theme is supported by the participants' responses quoted verbatim.

**Peer pressure:** participants stated that peers play a key role in decision-making, encourage risky behaviours and give misleading information. *"Under this working environment peers influence their friends' decision-making, especially young people are encouraged to have premarital sex, and they also attract older men" "Young women encourage each other to have sex outside marriage." "There is a growing number of people living with HIV and AIDS who engaged in unprotected sexual activity with HIV negative people, because they get misleading information from peers that when one have unprotected sex with a person who is not infected HIV can be cured"*. In addition, participants imply that lacking information, placing too much trust in peers and desiring to "fit in" and impress their friends are probable reasons why workers succumb to peer pressure. *"If a person do not have the right information, cannot say no sometimes friends will influence us." "Most young people depend too much on the trust they have in friends (because they grew up together) peer pressure justify their practice of unprotected sex and of not using condom" "Because of peer pressure regardless of the fear of HIV/AIDS, they (referring to some colleagues) are still afraid to buy their own condoms, afraid of what their friend will say"*.

**Communication:** participants in this study stated that (poor) communication influences HIV risk-taking behaviour by workers. Participants reported that they lack facts about HIV in their communities and this leads to risk-taking behaviour by workers. *"The lack of HIV facts information in our community results in people not to disclosed their HIV status and those who are not tested not go for voluntary testing"*. Furthermore, participants referred to poor parental communication about sex and HIV infection. *"Poor communications between young people and their parents on the subject of sex create a lot of problems. They are therefore forced to listen to friends." "Our parents do not talk about sex, HIV, or condoms frequently with their children because they considered these topics disrespectful, on the other side"*.

**Cultural norms:** workers mentioned community-held, socially shared cultural norms and beliefs on condom use, multiple sexual partners, gender inequality and unequal communication practices as factors that influence HIV risk-taking behaviours. Men's reluctance to use and their dislike for condoms, and the belief that only unfaithful woman, would request male partners to use condoms prevents women from practising safe sex. *"Married women who want to protect themselves are suspected to be unfaithful if they ask their husbands to use condoms." "In our community we are facing culture of continued reluctance to use condoms in sexual relationships"* Communities often revere men who have more than one partner. *"Cultural norms are condoning men to have different sexual partners and the same men are refusing to use condoms therefore contribute significantly to spreading HIV and are especially resilient to change in most cases"*. Community beliefs and cultural norms may perpetuate gender inequality (women are expected to be subservient to men) and shape patterns of communication within intimate relationships (women may not talk to their husbands about sex) [12]. *"I am also aware that culture has a role to play especially among women to make decisions, women cannot negotiate safe sex, the culture of paying lobola (pride price), proofs women's sexual obligation, gives men the power over women that they own them and therefore women cannot negotiates or decide when and how to have sex" "We don't talk to our husband about sex, it's our culture"*.

**Societal norms:** participants in this study cited fear of stigma and discrimination, particularly fear of social rejection by others, losing their jobs and being humiliated, as factors that promote HIV risk-taking behaviour.*"People still get fired from their jobs and people still get kicked out of their houses. Moreover, the fear is there even now most people are not able to disclose their HIV status to their partners. They do this to avoid rejection, refusals to have sex or of being stigmatized or discriminated against." "Community members may not seek testing because of the fear of discovering that they are HIV positive and fear of the resulting stigma and discrimination. Further, family members may encourage relatives with HIV/AIDS to remain silent about their HIV status to prevent gossip, social rejection and HIV-related stigma"*. This finding highlights the need for continued work in Lesotho to address HIV discrimination and stigmatisation. Factory workers who participated in this study report that low wages/income promote transactional sex by female factory workers. *"Transactional sexual relationships is encouraged somehow in our community because of the state of poverty as a result of insufficient income in the family. This leads to high rates of migration, now women left their homes and villages to care for families. These in most cases lead one to have sex partner relationship (transactional sexual relationship) in order to receive additional support for food, transport"*. Moreover, it has been reported that the majority of sex workers in the districts of Maseru and Leribe were originally textile workers who found sex work more profitable than the low wages offered by the factories [13].

## Discussion

**Figure 1:**
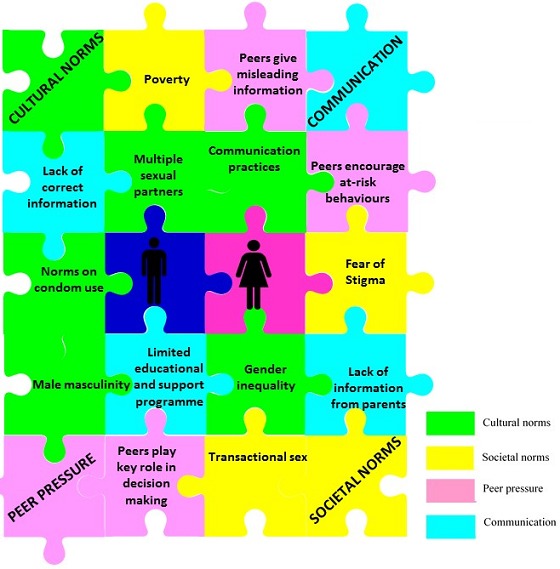
Schematic showing factors that influence HIV risk-taking behaviours amongst textile factory workers living with (TFWLWH) in Lesotho. The puzzle diagram depicts that these factors can be interdependent on each other. For example, lack of correct information in the community can influence cultural norms/myths on condom use. Similarly, peers that encourage at-risk behaviours may influence an individual to indulge in multi-partner and/or transactional sex that can also be precipitated by poverty

Efforts to curtail the spread of HIV have been directed along two distinct, strategic avenues: (a) preventing new infection and (b) early diagnosis and initiation of treatment in a maximum number of HIV-infected persons [14]. In a renewed effort to achieve the former, there are calls to use a positive approach that stimulates behaviour change among HIV seropositive individuals, so that they make decisions to take care of their health and prevent possible harm to others [15]. Factors this study found to influence HIV risk-taking behaviour amongst the workers are consistent with findings in literature [12, 16-21] Furthermore, these factors could be interdependent on one another as presented in the puzzle schematic in Figure 1.

## Conclusion

Whilst acknowledging the contribution made by current HIV prevention, treatment and support programmes for textile factory workers in Lesotho, this study confirmed the perception that TFWLWH in Lesotho indulge in HIV risk-taking behaviours. It is recommended that further investigations be carried out to establish if the factors associated with HIV risk-taking behaviours as identified by this study are present in the greater Basotho population. This should be done prior to proposing recommendations for decision-makers in health departments, community-based organisations and other stakeholders involved in the prevention of HIV and AIDS in Lesotho.

### What is known about this topic

- Lesotho has one of the highest HIV burdens in the world;
- Textile factory workers in Lesotho have the second highest HIV prevalence (42.7%) amongst worst affected groups.

### What this study adds

- This study confirmed the perception that TFWLWH in Lesotho indulge in HIV risk-taking behaviours;
- This study identified and contextualised factors associated with risk-taking behaviours amongst TFWLWH in Lesotho.

## Competing interests

The authors declare no competing interest.
